# An Energy-Efficient Secure Routing and Key Management Scheme for Mobile Sinks in Wireless Sensor Networks Using Deployment Knowledge

**DOI:** 10.3390/s8127753

**Published:** 2008-12-03

**Authors:** Le Xuan Hung, Ngo Trong Canh, Sungyoung Lee, Young-Koo Lee, Heejo Lee

**Affiliations:** 1 Dept. of Computer Engineering, Kyung Hee University, Seocheon, Giheung, Yongin, Gyeonggi, Korea; E-Mails: ntcanh@oslab.khu.ac.kr; yklee@khu.ac.kr; 2 Dept. of Computer Science and Engineering, Korea University, Anam-dong, Seongbuk-gu, Seoul 136-713, Korea; E-Mail: heejo@korea.ac.kr

**Keywords:** Secure routing, key management, sensor networks, sink mobility, deployment knowledge

## Abstract

For many sensor network applications such as military or homeland security, it is essential for users (sinks) to access the sensor network while they are moving. Sink mobility brings new challenges to secure routing in large-scale sensor networks. Previous studies on sink mobility have mainly focused on efficiency and effectiveness of data dissemination without security consideration. Also, studies and experiences have shown that considering security during design time is the best way to provide security for sensor network routing. This paper presents an energy-efficient secure routing and key management for mobile sinks in sensor networks, called SCODE*plus*. It is a significant extension of our previous study in five aspects: (1) Key management scheme and routing protocol are considered during design time to increase security and efficiency; (2) The network topology is organized in a hexagonal plane which supports more efficiency than previous square-grid topology; (3) The key management scheme can eliminate the impacts of node compromise attacks on links between non-compromised nodes; (4) Sensor node deployment is based on Gaussian distribution which is more realistic than uniform distribution; (5) No GPS or like is required to provide sensor node location information. Our security analysis demonstrates that the proposed scheme can defend against common attacks in sensor networks including node compromise attacks, replay attacks, selective forwarding attacks, sinkhole and wormhole, *Sybil* attacks, HELLO flood attacks. Both mathematical and simulation-based performance evaluation show that the SCODE*plus* significantly reduces the communication overhead, energy consumption, packet delivery latency while it always delivers more than 97 percent of packets successfully.

## 1. Introduction

Wireless Sensor Networks (WSNs) comprises of a large number of sensor nodes that are densely deployed either inside the phenomenon or very close to it. In sensor networks, a source is defined as a sensor node that detects a stimulus, which is a target or an event of interest, and generates data to report the stimulus. A sink is defined as a user collecting these data reports from the sensor network. In many sensor network applications such as military, homeland security or environmental surveillance, it is necessary for users (sinks) to access sensor networks while they are moving. For example, in a battle- field a soldier with a PDA in hand might continuously collect an enemy tank's movement information while he is moving.

Sink mobility brings new challenges to secure routing in large-scale sensor networks. To maintain a dissemination rout, a mobile sink has to constantly propagate its current location information to all sensor nodes. That introduces significantly communication overhead. Furthermore, intermediate nodes on the new rout have to discover their neighbors and exchange secret information to establish a secure communication. That also introduces more computation and communication overhead.

A number of routing protocols have been proposed for WSNs such as LEACH [1], SecRout [2], SEEM [3], SeRINS [4], TTSR [23]. However, these works do not take into account of sink mobility and their routing paths remains static. So they are not suitable to work in such mobile sink circumstances. Several studies have considered the scalability and efficiency of data dissemination from multiple sources to mobile sinks such as TTDD [17], Directed Diffusion (DD) [18], SAFE [5], SEAD [6], Declarative routing protocol [7], and GRAB [8]. However, the authors mainly focused on energy efficiency. These approaches are very vulnerable from many attacks in sensor network routing such as spoofed, altered, or replayed routing information [9][16], selective forwarding, sinkhole [16], *Sybil* [10], wormhole [11], and HELLO flood (unidirectional link) attacks [16]. Also, most of existing routing protocols consider routing protocols and security schemes (like key management) separately. As a consequence, it is unsurprising that they are vulnerable to many attacks. It is not trivial to fix the problem since it is unlikely that a sensor network routing protocol can be made secure by incorporating security mechanisms after the design has completed. Studies and experiences have shown that considering security during design time is the best way to provide security and efficiency for sensor network routing.

In this paper, we propose an energy-efficient secure routing protocol and key management scheme for mobile sinks in sensor networks, called SCODE*plus*. This scheme is a significant extension of our previous work [12][26] in five aspects:

(1) Key management scheme and routing protocol are considered during design time to increase security and efficiency. In our previous study, we focused on the efficiency for the secure routing protocol with an assumption that the sensor network achieves a key infrastructure from LEAP+ [14].
(2) The network topology is organized in a hexagonal plane. Compared with our previous square-grid topology, the hexagonal topology brings more efficiency. It decreases the number of cells, reduces transmission hops, and increases the number of sleeping nodes to save more energy. This is because the hexagonal geometry provides the best approximation to circle and covers the biggest area as compared to the rectangle or triangle. Also, a hexagon has the least neighboring cells (six) compared with a rectangle (eight) or a triangle (twelve).
(3) The key management scheme can eliminate the impacts of node compromise attacks on links between non-compromised nodes. Our previous work has a weakness with impacts of nodes compromise attacks like most existing key management schemes.
(4) Sensor node deployment conforms to *Gaussian* distribution. In our previous study, we assumed that all sensor nodes are uniformly distributed. However, this is not realistic and it might raise unexpected problems when we deploy the protocol in reality.
(5) No GPS (*Global Position System*) or like is required to provide node's location information. In SCODE, we have assumed that sensor nodes need to be aware of their locations. However, attaching GPS or like increases high cost for sensors, and thus reducing the feasibility of the deployment. By using deployment knowledge, the proposed scheme can establish a key infrastructure and end-to-end communication for the sensor network.

The major contributions of this study are then four fold:

(1) We propose a first, novel secure routing protocol and key management scheme supported sink mobility in WSNs.
(2) Compared with existing approaches, the proposed routing protocol is more energy-efficient, lower communication overheads, high packet delivery ratio (>97%), and secure against the common attacks in sensor networks.
(3) Our key management scheme takes advantage of the hexagonal topology and expected location information not only to reduce the memory cost but also get better resilience against node compromise attacks. Moreover, the scheme can eliminate the impacts of node compromise attacks on links between non-compromised nodes.
(4) Intensive theoretical as well as simulation-based analysis and evaluation studies show that the proposed scheme achieves better security and efficiency than our previous work as well as other popular approaches like TTDD and DD.

The rest of the paper is organized as follows: Section 2 discusses common attacks in sensor networks routings. Section 3 contains brief description on the GAF protocol, as it is used as an underline protocol in our proposal. Section 4 briefly describes our previous study SCODE. Section 5 presents SCODE*plus* scheme in details including key management scheme and routing protocol. We analyze security and cost of the scheme in Section 6. Section 7 shows performance evaluation results. Section 8 discusses some existing routing protocols as well as their vulnerabilities and shortcomings. Finally, we conclude the paper in Section 9.

## 2. Attacks on Sensor Network Routing

Attacks on sensor network routing have been discussed in several papers [9][10][11][16]. Most of the attacks fall into one of the following categories: spoofed, altered, or replayed routing information [9][16], selective forwarding [16], sinkhole [16], *Sybil* [10], wormhole [11], and HELLO flood (unidirectional link) attacks [16]. We briefly describe those attacks on sensor networks:

○ ***Spoofing, altering, or replaying routing information***[9, 16]: By spoofing, altering, or re-playing routing information, the adversaries can create routing loops, attract or repel network traffic, extend or shorten source routes, generate false error messages, partition the network, increase end-to-end latency, etc.
○ ***Selective forwarding attacks***[16]: Malicious nodes may refuse to forward certain messages and simply drop them, ensuring that they are not propagated any further. Another form of this attack is that an adversary selectively forwards packets, i.e. she is interested in suppressing or modifying packets originating from a few selected nodes, reliably forwards the remaining traffic and limits suspicion of her wrongdoing.
○ ***Sinkhole and wormhole attacks***[16]: In sinkhole attacks, an adversary attracts nearly all the traffic from a particular area through a compromised node, creating a metaphorical sinkhole with the adversary at the center. In wormhole attack, an adversary tunnels messages received in one part of the network over a low latency link and replays them in a different part. Wormhole attacks more commonly involve two distant malicious nodes colluding to understate their distance from each other by relaying packet along and out-of-bound channels available only to the attacker.
○ ***Sybil attacks***[10]: In *Sybil* attacks, a single node presents multiple identities to other nodes in the network. In particular, a Sybil attack causes a significant threat to geographic routing protocols. Using a Sybil attack, an adversary can cheat as many nodes at the different locations.
○ ***HELLO flood (unidirectional link) attacks***[16]: A laptop-class attacker may broadcast routing or other information with large enough transmission power and convinces every node in the network that the adversary is its neighbor. As a consequence, these nodes only relay packets to the attacker's laptop.

## 3. Geographical Adaptive Fidelity (GAF)

In our protocol, sensor nodes within a cell periodically negotiate among each other to elect the coordinator in every round. For each round, only one node stays active to be a coordinator, while the others fall into sleeping mode. Doing this significantly reduces the energy consumption because nodes in the idle state spend much more energy as compared with the sleeping state. Analysis in [31] has shown that energy consumption ratio for *sleep:idle:receive:transmit* is 0.13:0.83:1:1.4. It also reduces the network congestion because the number of nodes participating in transmission/reception is decreased. On the other hand, frequent change of coordinator role helps the particular nodes not running out of its energy quickly. Therefore, it can prolong nodes as well as the network lifetime. In order to control nodes in different states and transition, we employ *Geographical Adaptive Fidelity* (GAF) protocol [15]. The GAF conserves energy by identifying nodes that are equivalent from a routing perspective and then turning off unnecessary nodes, keeping a constant level of routing *fidelity*.

### 3.1. Determining node equivalence

Even with location information, it is not trivial to find equivalent nodes in a network. Nodes that are “equivalent” between some nodes may not be equivalent for communication between others.

**Figure 1.**
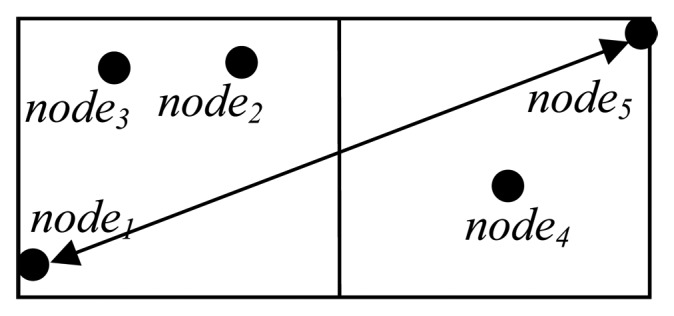
Example of virtual grid in GAF.

To address this issue, the GAF uses location information and virtual grids to determine node equivalent. Two nodes are equivalent if they locate in the same virtual cells. The size of each virtual cell is determined based on the nominal radio range *R*. Assume that the virtual grid is a square with *r* units on a side as show in Figure 1. In order to meet the definition of virtual cell, the distance between two possible farthest nodes in any two adjacent cells, must not be larger than *R*. For example, *node_1_* and *node_5_* in Figure 1 are at the end of the long diagonal connecting two adjacent cells. Therefore, we get *r*^2^ +(2*r*)^2^ ≤ *R*^2^ or $r\leq R/\sqrt{5}$.

### 3.2. GAF state transitions

**Figure 2.**
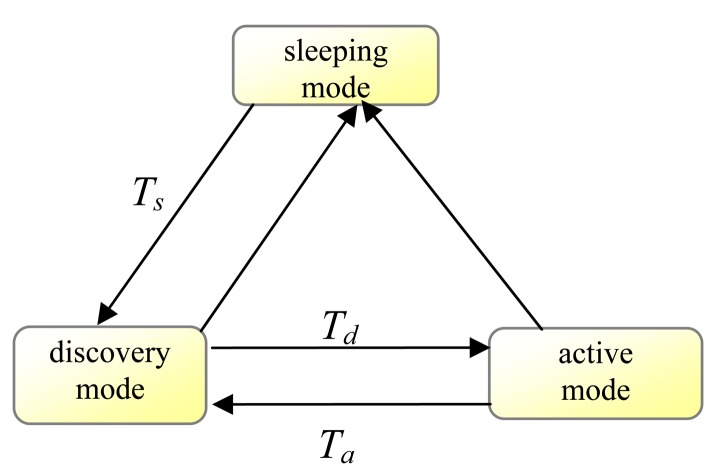
State transitions in GAF.

In the GAF, nodes are in one of three states: *sleeping*, *discovery*, *active*. A state transition diagram is shown in Figure 2.

Initially nodes start out in the discovery state, in which a node turns on its radio and exchanges discovery message to find other nodes within the same cell. The discovery message is a tuple of node ID, cell ID, *estimated node active time* (*enat*), and node state. As described above, a node uses its location and cell size to determine the cell ID.

When a node enters discovery state, it sets a timer for *T_d_* seconds. When the timer fires, the node broadcasts its discovery message and enters state active. The timer can also be suppressed by other discovery messages. This timer reduces the probability of discovery message collision. When a node enters active, it sets a timeout value *T_a_* to define how long this node can stay in active state. After *T_a_*, the node will return to the discovery state. While active, the node periodically re-broadcast its discovery messages at an interval of *T_d_*.

A node in discovery or active states can change state to sleeping when it can determine some other equivalent nodes will handle routing. When transitioning to sleeping, a node cancels all pending timers and powers down its radio. A node in the sleeping state wakes up after an application-dependent sleep time *T_S_* and transitions back to discovery.

The GAF leaves choices of many parameters including *enat*, *T_d_*, *T_a_*, node tank, *T_s_* to application. Applications may wish to optimize these choices, for example, perhaps trading increased packet loss for greater energy savings. *enat* can be set to the expected node lifetime, conservatively set by assuming the node will constantly consume energy at a maximum rate until it dies. GAF chose *T_d_* as a uniform random value between 0 and some constant. This approach avoids contention from synchronized discovery message. The GAF uses *T_a_* to accomplish load balancing. *Node ranking* in GAF is chosen to maximize network lifetime by selecting which nodes handle routing. Rank is determined by several rules. For example, nodes with longer expected lifetime higher rank. GAF employs a load balancing strategy so that all nodes remain up and running together for as long as possible.

## 4. SCODE – Primarily Proposed Secure Routing Protocol

### 4.1. Overview

Before presenting our enhanced security scheme, we briefly describe the basic scheme *Secure COordination-based Data dissEmination* protocol (SCODE) [12]. The SCODE is based on the following assumptions.

(1) There may be hundreds or thousands of homogeneous nodes uniformly distributed into a field.
(2) All sensor nodes are stationary and aware of their locations. Sensors may use GPS or a secure location discovery service, e.g. [13], to estimate their locations.
(3) Each node/sink has a unique ID and maintains three key types based on LEAP+ scheme [14]: a unique individual key *K_A_* that each node A shares with a sink; a cluster key *K_CH_* shared among all nodes in the same cell; and a pair-wise shared key *K_AB_* shared between a node/sink *A* and its neighbor *B* (Node B is defined as a neighbor of node A if and only if B coexists in the same or adjacent cell of A).
(4) Due to resource constraints, sensor nodes are not equipped with tamper-resistant hardware. If an adversary successfully compromises a sensor, then she can obtain all key material, data, and code stored on that node.
(5) Sinks are powerful nodes, moving within the sensor network field. Sinks are also aware of its location. Since sinks are mobile, sensors cannot know sink locations.
(6) Each sink stores a table containing (node, key) pairs (*ID_node_*, *K_node_*) of all the sensor nodes.
(7) Sinks are trusted.

**Figure 3.**
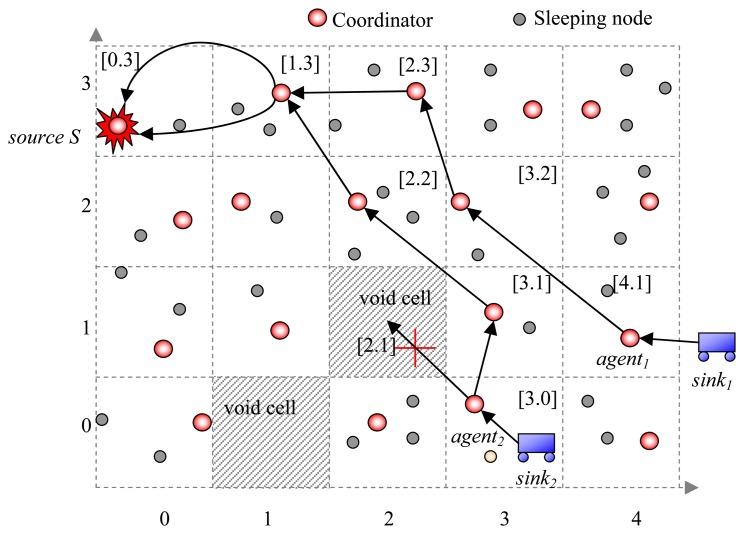
Routing in SCODE.

In SCODE, the network plane is partitioned into a virtual grid as illustrated in Figure 3. Each node can compute its cell ID [X,Y] as *X* = ⌊*x/r*⌋,*Y* = ⌊*y/r*⌋, where *r* is the cell size and (x,y) is node coordinate. Nodes in each cell negotiate based on GAF [15] so that there is only one node, called coordinator, stays awake to handle routing while the others fall into sleeping mode for the sake of energy saving. Since each cell has eight neighboring cells, the cell size should be appropriately determined so that any two nodes in adjacent cells could communicate with each other within one-hop transmission. For that reason, it must be (*2r*)^2^+(*2r*)*^2^*≤*R^2^* or $r\leq R/\sqrt{8}$, where *R* is radio range of sensor nodes.

After an interval, sleeping nodes wake up and elect again. When a stimulus appears, sensors surrounding it collectively process signal and one of them becomes a source to generate data reports. SCODE has three major phases: *data announcement*, *query transfer*, and *data dissemination*.

In the *data announcement* phase, the source broadcasts data announcement messages encrypted by its individual key to all coordinators. When a sink receives a data announcement message from a coordinator, it builds a query message, encrypted by the source's individual key and sends to the source. The query message is relayed through coordinators towards the source. During the *query transfer*, a routing path is established between the sink and the source. Upon receiving the query, the source starts data dissemination process to the sink. It generates data report and sends to the sink along the routing path.

The sink frequently checks its current cell ID. If it moves out of the current cell, it first sends a *cache-removal* message to remove the old path. Then it sends a new query to the source to update its new location and establishes a new routing path.

In order to detect a compromised or malfunctioning node, we introduce an *inspecting system*. In the inspecting system, all eight neighboring coordinators of each coordinator play roles of inspectors. The inspectors observe messages sent out from the inspected node and detect whether it is a compromised or malfunctioning node. For example, if a malicious node *B* receives a message from a node *A* and attempts to modify it before forwarding to *C*, the common inspectors of *A* and *B* will detect the change because they also receive the same messages sent out from *A* and *B*, so that they know the message originated from B is not the same as the original message sent out from *A*.

Analysis and simulation results in [12] have shown that SCODE is much more energy-efficient than existing approaches such as TTDD [17] and DD [18] while it always delivers 90% of packets to the mobile sinks successfully. More importantly, SCODE is secure against common attacks in sensor network routing mentioned in Section 2: spoofed, altered, or replayed routing information, selective forwarding, sinkhole, *Sybil*, wormhole, and *HELLO* flood (unidirectional link) attacks. Though SCODE is dominant over existing routing protocols, it still has a number of limitations, as pointed out in Section 1.

## 5. SCODE*plus*: Enhanced Secure Routing and Key Management Scheme

### 5.1. Hexagonal Network Deployment

In our proposal, the target area is divided in a hexagonal grid. This model is more practical in realistic scenarios. Sensor nodes in each group are delivered together, such as using aircraft to drop groups in sequence, so expected adjacent groups have better chance of being close to each other on the ground. Based on different deployment methods, the deployment distributions follow some specific probability distribution functions (*pdf*). The *pdf* may be a uniform distribution [27] or two-dimensional *Gaussian* distribution [28]. In this paper, for the sake of simplify in analysis, we use a *Gaussian* distribution, which is also widely studied and used in practice. Other distributions could be applied as well.

We first create the hexagonal topology and determine center points of hexagons on the geographical map of the area to be deployed. Then, we map center points to the real area. Supposed a sensor networks contains *N* nodes. We assume that there are *G* cells totally. Now we split ⌊*N/G*⌋ *G nodes into *G* groups. Each group will be deployed within a cell with ⌊*N/G*⌋nodes. Each of the remainder *N*-⌊*N/G*⌋ *G nodes is assigned to either random cells or any cells we would like to. After that, each group is one-by-one drop from the aircraft targeting to the predetermined deployment point. When the deployment point of group *G_i_* is at $\left(x_{i}^{o},y_{i}^{o}\right)$, the *pdf* for a node *n_i_* belongs to group *G_i_* is the following:

(1) $$f(n_{i}(x_{i},y_{i})|n_{i}\in G_{i})=\frac{1}{2\pi \sigma^{2}}e^{-[{\left(x_{i}-x_{i}^{o}\right)}^{2}+{\left(y_{i}-y_{i}^{o}\right)}^{2}]/2\sigma^{2}}=f(x_{i}-x_{i}^{o},y_{i}-y_{j}^{o})$$

where (*x_i_*,*y_i_*) is the coordinate of node *n_i_* in the group *G_i_* and *σ* is the standard deviation of the distribution. The value of *σ* depends mainly on the height of aircraft when dropping sensor groups.

Based on Gaussian distribution property, 99.87% nodes reside within an area with radius of 3σ. According to our simulation result, that ratio is 98.89%, which is almost similar. That means around 1.11% nodes resides outside the cell, it will not effect to the routing. Their positions are not far from the expected cell. Even in some case, those error nodes act as coordinators, it is still considered as the node in the expected cell, not belong to another cell.

**Figure 4.**
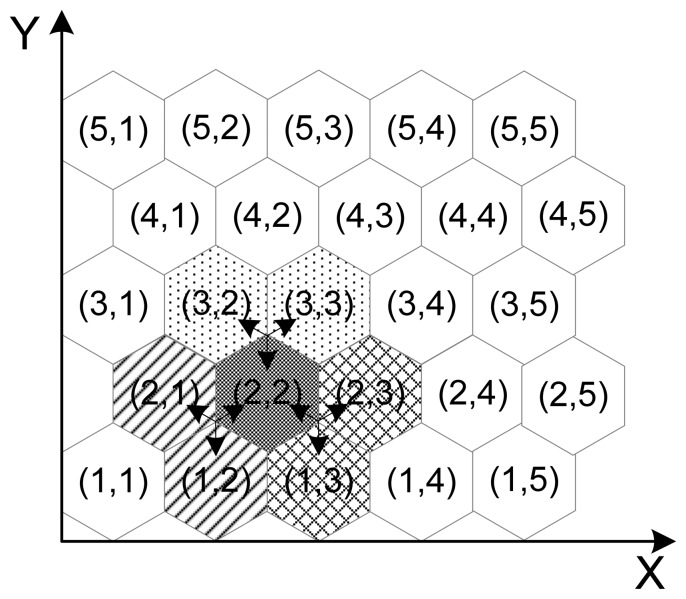
Hexagonal group-based deployment model.

We define a cluster is a set of three adjacent groups. There are three types of cluster. At any group *G_i,j_*, there are 1-cluster containing *G_i,j-1_* and G*_i-1,j_*, 2-cluster containing *G_i,j_*_+_*_1_* and *G_i-1;j_*_+_*_1_*, and 3-cluster containing *G_i_*_+_*_1,j_* and *G_i_*_+_*_1,j_*_+_*_1_*. For example in Figure 4, the cell [2,2] has 1-cluster cells [2,1] and [1,2], 2-cluster cells [2,3] and [1,3], and 3-cluster cells [3,2] and [3,3].

### 5.2. Key Management

Our key management scheme is an improvement of Blundo's scheme [29]. Bludo's scheme is not able to apply directly to sensor networks due to its memory overhead for storing keys. It uses *n* symmetric variables polynomials with *t-degree* to establish key distribution for *t-secure n-conference*. In this scheme, each node must store *t*+*1* coefficients. Each coefficient costs *log_2_q* bits. So the memory storage requirement for each node in this model is (*t*+*1*)*log_2_q* bits. The analysis in [29] shows that, this scheme is unconditionally secure and *t-collusion* resistant. It means that as long as no more than *t* nodes are compromised, the attacker knows nothing about the pair-wise key between any two non-compromised nodes. However, the size of memory depends exponentially on the size of the network, so it is not useful for such resource-constraint devices like sensor nodes using only this model. We have tackled this problem by using pre-deployment knowledge and showed that it will take more advantages than other polynomial-based schemes applied location knowledge. The previous work [26] has demonstrated our success in solving the problem using the square-grid network topology. In this study, a new effort is made to enhance the scheme with a hexagonal topology.

We define a key-space as derived from a bivariate polynomial in Blundo's scheme. A node *N_A_* picks a key-space *f_u_*_,_*_v_*(*x*, *y*) if it carries the coefficients of *f_u_*_,_*_v_*(*N_A_*⊕ *nonce_A_*, *y*), where *nonce_A_* is a random value of node *A* and will be described in the *key predistribution* phase. When two nodes are in the same key-space, they could calculate a pair-wise shared key to setup a secure channel.

Our scheme allows sensor nodes to find a common key space with each of their neighbors after deployment. It consists of three phases: *key predistribution, direct key establishment*, and *indirect key establishment*. The key predistribution phase is carried out to preload the credential information to each sensor node before deployment. After setting up, two sensor nodes can establish a direct key between them if they share at least a common key-space, otherwise, they could agree on an indirect key according to the indirect key establishment phase.

#### 5.2.1. Key Predistribution Phase

The purpose of this phase is to assign key materials to each node. Based on these key materials, neighboring nodes could setup pair-wise keys after deployment.

**Figure 5.**
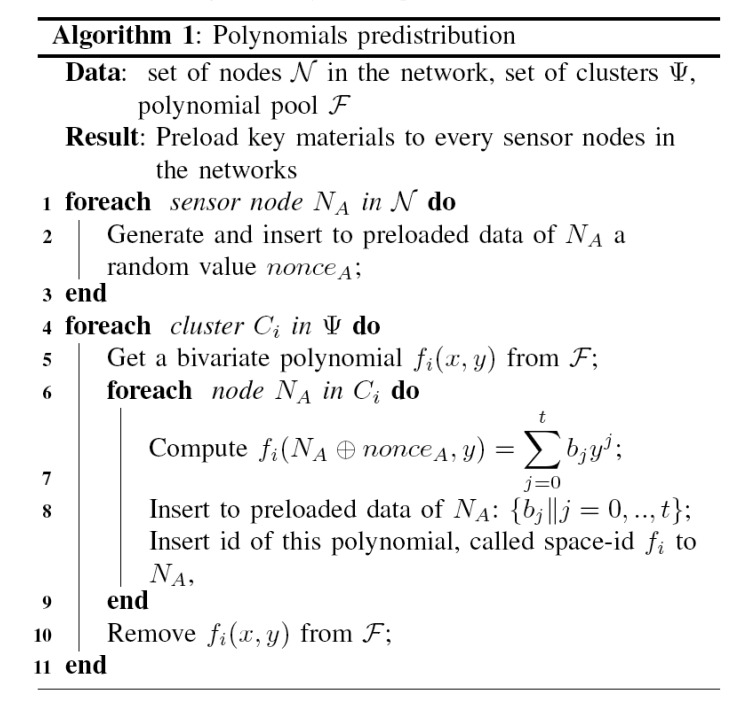
Polynomials predistribution.

This task is done by an offline server. At first, the server will generate a polynomial pool *F* containing enough *t-degree* symmetric bivariate polynomials for every cluster. Then it distributes each polynomial to all sensor nodes in each cluster. Since each cell belongs to three clusters, therefore every node has to store knowledge of three *t-degree* bivariate polynomials. In other words, each node needs to pick three key-spaces. The detail algorithm for polynomials predistribution is shown in Figure 5.

At the end of this phase, every sensor node stores node IDs, three space IDs, random values, and three vectors of coefficients equivalent to three key-spaces. These key materials will be used to setup pair-wise keys in the next phase.

#### 5.2.2. Direct Key Establish Phase

After deployment, every sensor node discovers the sharing key-space with its neighbors. Assume that node *N_A_* with three space IDs *f_i_*, *f_j_*, *f_k_* needs to discover shared key-space with its neighbors. It broadcasts a 1-hop discovery message *Key-Space Discovery Message* (KSDM) containing the following information:

$$N_{A},\text{nounc}e_{A},H(f_{i}⊕\text{nounc}e_{A}),H(f_{j}⊕\text{nounc}e_{A}),H(f_{k}⊕\text{nounc}e_{A})$$

where *H* is the hashing function and ⊕ is the XOR operation.

When a neighbor of *A*, say *B*, receives this message, it finds out that it could share three, one or no common key-space with *A*. Similarly, node *A* also receives *B*'s KSDM message and finds out common key-spaces. If the sharing is at least one common key-space, the pair-wise key between *B* and *A* is calculated at B as follows:

(2) $$K_{AB}=f(N_{B}⊕\text{nounc}e_{B},N_{A}⊕\text{nounc}e_{A})$$

After getting *K_BA_*, node *B* deletes value *nonce_A_* from its memory. The process of computing pair-wise key at *A* is similar. Because of the symmetric property of bivariate polynomials, *K_AB_* = *K_BA_*. After this phase, every node stores a list of pair-wise keys with its neighbors, beside the key-space information and a random value in previous phase.

#### 5.2.3. Indirect Key Establishment Phase

In case there is no common key-space between two neighboring nodes, it is needed to establish a path key through one or more intermediate nodes. Our solution for this problem is as follows.

After the *direct key establishment* phase, every node *A* knows a set of secure neighboring nodes, denoted as *S_A_*. Node *A* wants to establish a pair-wise shared key with its neighbor *B*, but *B* and *A* do not share any key-space. In this situation, *A* generates a session key, called *K_S_*, and find a node say *C* in *S_A_* that have the same group ID with node *B* or neighboring group ID of group containing node *B*. Node *A* then sends a message containing *K_S_* encrypted by key *K_AC_* to node *C*. In turn, node *C* sends to *B* a session key through a secure channel protected by the key *K_CB_*. The key *K_S_* then is used as pair-wise shared key between two nodes *A* and *B*.

After above three phases, every node stores a table containing neighbor IDs and pair-wise shared keys equivalently. The existence of key materials allows sensor networks to be able to add new nodes for replacement later.

#### 5.2.4. Establishing/Revoking Keys of New/Existing Sensors

To add a new sensor, the key setup server only needs to predistribute the related polynomial shares to the new node, similar to predistribution phase. Since the size of key-space is limited, the more sensors are added, the lower the security in that cell becomes.

The revocation method is also straightforward. Each sensor node only needs to store a black list IDs of compromised sensors that share at least one bivariate polynomial with itself. If there are more than *t* compromised nodes sharing the same polynomial, the non-compromised nodes that have this polynomial will remove this polynomial and all related compromised nodes.

### 5.3. Secure Data Dissemination Scheme to Mobile Sinks

In this section, we present SCODE*plus*, a significantly enhanced version of the SCODE [12]. Different from the previous version, the routing algorithm in SCODE*plus* does not require locations of sensor nodes. Each node knows their own group ID (cell ID) prior to the deployment. SCODE*plus* also has three phases: *data announcement, query transfer*, and *data dissemination*. Those phases are similar to that of the SCODE except the routing algorithm in *query transfer* phase. The previous routing algorithm is based on square-grid structure. In this version, the routing algorithm is based on the hexagonal network topology which provides more straight routing path between a source and a sink. On the other hand, thank to our enhanced deployment model, the new routing algorithm can eliminate the void cell problem of the previous scheme.

Based on our improvement in this version, we lessen the assumptions 1, 2, and 3 mentioned in Section 4.1 as follows:

(1*) There may be hundreds or thousands of homogeneous nodes distributed into a sensor field. The distribution of nodes within a cell conforms to *Gaussian*. This is more realistic.
(2*) All sensor nodes are stationary and NOT aware of their locations.
(3*) Each node/sink has a unique ID and maintains three key types based on our key management scheme: a unique individual key *K_A_* that each node *A* shares with a sink; a cluster key *K_CH_* shared among all nodes in the same cell; and a pair-wise shared key *K_AB_* shared between a node/sink *A* and its neighbor *B* (Node B is defined as a neighbor of node A if and only if B coexists in the same or adjacent cell of A). Those keys can establish through *direct key establish* phase or the *indirect key establish* phase.

Table 1 provides the notation description which will be used in the paper.

#### 5.3.1 Secure Neighboring Discovery

After deployment, each node *A* broadcasts a *HELLO* message in order to discover its neighborhoods. This message is encrypted by *A*'s cluster key *K_CH_*:

$$A\to \text{broadcast}:\left\{ID_{A}|CID_{A}|N_{0}\right\}K_{CH}$$

Each receiving node *B* decrypts the message and checks if it is a neighbor of *A*. If yes, it replies to *A* node ID and cell ID along with a *nonce* value *N_0_* encrypted by the pair-wise shared key *K_AB_*.

$$B\to A:\left\{ID_{B}|CID_{B}|N_{0}+1\right\}K_{BA}$$

Receiving node *A* decrypts the message using the pair-wise shared key. It then checks if the *nonce N_0_* is the one it has broadcasted. If it is, *A* accepts *B* as a neighbor and updates its neighborhood table.

The above two-way handshake protocol can avoid (or defend against) the unidirectional link problems (or attacks) [16]. For example, if an attacker uses node *A* which is a more powerful node such as a laptop with longer transmission range than *B*, then *A* can send a message to *B* directly, but *B* cannot send a message to *A* within one-hop. However, node *B* still thinks that *A* is a one-hop neighboring node and various problems may arise. For example, *B* tries to packets to *A*, since A is out of *B*'s communication range, the packets will be dropped.

#### 5.3.2. Three Main Phases

**Figure 6.**
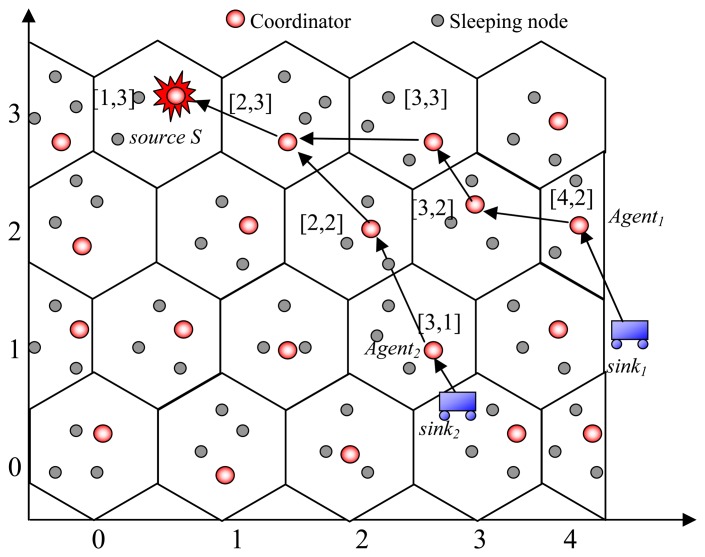
An example of routing in SCODE*plus*.

(a) Phase 1: Secure Data Announcement:
  When a stimulus is detected, a source *S* propagates a *Data-Announcement* (DA) message to all coordinators using a flooding mechanism. The message contains source ID, cell ID and a MAC:
  $$\begin{array}{ll} S\to \text{broadcast}: & \text{PID}|ID_{S}|CID_{S}\left\{\text{DA}\right\}K_{S}|\text{MAC}(K_{S},\text{PID}|ID_{S}|CID_{S}|\left\{\text{DA}\right\}K_{S}) \end{array}$$
  Every coordinator stores a few piece of information for the route discovery, including the information of the stimulus and its cell ID. Since the coordinator role might be changed every time, cell ID is the best solution for nodes to know the target it should relay the query to. In order to avoid indefinite storing of data-announcement messages in each coordinator, the source attaches a *timeout* parameter in each message. Within the timeout interval, if the coordinator has not received any further data-announcement message, it removes the information of the stimulus and the source location to free the cache.
(b) Phase 2: Secure Query Transfer and Route Discovery
  Receiving a data-announcement message, a sink looks up in its {(*ID_node_*, *K_node_*)} table, finds the key *K_S_* shared with the source, and uses it to decrypt the message. It then constructs a query message using *K_S_* and sends back to the source via coordinators as follows:
  ○ The sink first send message to its agent. The agent is a coordinator within the same cell. If the sink locates outside of the network (not belong to any cell), then the agent would be the coordinator in the closest cell.
    $$\begin{array}{ll} \text{Sink}\to \text{Agent}: & \text{PID}|CID_{\text{This}}|CID_{\text{Next}}|ID_{s}|CID_{S}|ID_{\text{Sink}}|\left\{\text{QUERY}\right\}K_{S}|\text{MAC}(K_{\text{SinkAgent}},\text{PID}|CID_{\text{This}}|CID_{\text{Next}})|\text{MAC}(K_{S},ID_{S}|CID_{S}|ID_{\text{Sink}}|\left\{\text{QUERY}\right\}K_{S}) \end{array}$$
    where *CID_This_* is cell ID of the agent, and *CID_Next_* is cell ID of the next cell (in this case *CID_This_*= *CID_Sink_*, and *CID_Next_* = *CID_Agent_*). The source key *K_S_* is used to encrypt the query content. It is also used to build a *MAC* of *ID_S_*, *CID_S_*, and *ID_Sink_* in order to provide data authentication and data integrity of the information sent from the sink. The pair-wise shared key *K_SinkAgent_* is used to build a *MAC* of *PID*, *CID_This_*, and *CID_Next_* in order to provide data authentication and data integrity of the packet sent from the current node.
  ○ The agent computes next cell ID (which is the closest cell to the destination) towards the source, and then forwards the packet to it.
    $$\begin{array}{ll} \text{Agent}\to \text{Nextcell}: & \text{PID}|CID_{\text{This}}|CID_{\text{Next}}|ID_{s}|CID_{S}|ID_{\text{Sink}}|\left\{\text{QUERY}\right\}K_{S}|\text{MAC}(K_{\text{AgentNextl}},\text{PID}|CID_{\text{This}}|CID_{\text{Next}})|\text{MAC}(K_{S},ID_{S}|CID_{S}|ID_{\text{Sink}}|\left\{\text{QUERY}\right\}K_{S}) \end{array}$$
  ○ Receiving coordinator checks whether its cell ID is the same as *CID_Next_* in the message. If yes, it computes next cell ID, and relays the message to it. Each node maintains a routing table which stores the departure cell ID, the destination cell ID, the uplink cell ID (which it receives the message) and the downlink cell ID (which is next cell ID *CID_Next_*).
  An example is illustrated in Figure 6. The *sink_1_* first sends a query to its agent in cell [4,2]. The coordinator in [4,2] looks up its neighboring table and finds out that cell [3,2] is the closest one to the source *S* in cell [1,3]. Similarly, the query is forwarded to the *S* through the path {*sink_1_*, [4,2],[3,2],[3,3],[2,3], *S*}. Likewise, the query from the *sink_2_* is relayed through the path {*sink_2_*, [3,1],[2,2],[2,3], *S*}. Each intermediate node maintains an uplink table towards the sinks for data dissemination afterwards. At cell [2,3], the coordinator receives the same query from different sinks, so it stores only one query for two sinks. Later on, only one DATA packet is sent from source to [2,3] for both sinks, instead of the two packets. At [2,3], the coordinator check its query cache and generate two DATA packets and sends to uplink [3,3] and [2,2] towards *sink_1_* and *sink_2_*, respectively.
(c) Phase 3: Secure Data Dissemination
  When a source receives a query from a sink, it starts generating reports. Data messages are encrypted by using the source key *K_S_*. The source first send to its uplink node *A*:
  $$\begin{array}{ll} S\to A: & \text{PID}|CID_{\text{This}}|CID_{\text{Next}}|ID_{s}|CID_{S}|ID_{\text{Sink}}|\left\{\text{DATA}\right\}K_{S}|\text{MAC}(K_{SA},\text{PID}|CID_{\text{This}}|CID_{\text{Next}})|\text{MAC}(K_{S},ID_{S}|CID_{S}|ID_{\text{Sink}}|\left\{\text{DATA}\right\}K_{S}) \end{array}$$
  Receiving node *A* checks if the data packet is forwarded to itself or not by comparing its cell ID with *CID_Next_* in the packet. If not, it keeps the packet for a short time for inspecting purpose before dropping it. Otherwise, it computes next cell ID, changes *PID*, *CID_This_*, *CID_Next_* and compute a new MAC to replace MAC (*K_SA_*, *PID* | *CID_This_* | *CID_Next_*). After that, it relays the packet to the next cell. When the message reaches the sink, it verifies MAC value in the message to ensure its data authentication and integrity. It then decrypts the message by using *K_S_*.

#### 5.3.3. Sink Mobility Management

Periodically, the sink checks the distance to the agent's cell. If it recognizes that it no longer reaches that cell within the sensor transmission range, the sink has to compute new cell ID and selects a coordinator in that cell as a new agent. Then, the sink re-sends a query to the source to establish a new data dissemination route by using the same mechanism described in the phase 2. By re-sending queries only when the sink moves out of transmission range, SCODE*plus* significantly reduces communication overhead compared with other approaches. Hence, collision and energy consumption are reduced. Also, the number of loss data packet is decreased.

#### 5.3.4. Coordination Election

As aforementioned, the proposed scheme is based on the GAF to establish a coordination network. During the discovery phase, a node with the highest ranking will stay awake and play a role of a coordinator while the others fall into sleeping mode. The node ranking is determined by an application-dependent ranking procedure (it can be an arbitrary ordering of nodes to decide which nodes would be active, or it can be selected to optimize overall network lifetime). In GAF, a node with a longer expected lifetime is assigned a higher rank. This rule put nodes with longer expected lifetime into use first. However, this is very vulnerable. An adversary can use a compromised node to advertise the highest rank so that this node can be active all the time.

Practically, it is very hard (perhaps not possible) to absolutely defend against node compromise attacks for GAF. Therefore, we modify the discovery process and the ranking rule of GAF in order to reduce the risk without lessening its advantages. A coordinator is orderly selected according to node ID at each discovery round. For example, there are *m* nodes {*ID_1_*, *ID_2_*,…, *ID_m_*} (*ID_1_*< *ID_2_*< … < *ID_m_*) in a certain cell, if node *ID_i_* is a coordinator at the current round, then *ID_i_*_+_*_1_* would be a coordinator at the next round; if *i* = *m* then “*i* + 1” would be 1. By doing this way, the adversary cannot make her node active all the time at will. The compromised node is active only if it is a coordinator at the given round. On the other hand, our enhancement reduces communication overhead compared with GAF since nodes do not need to broadcast discovery messages.

Once a new coordinator is elected, the old coordinator forwards all current routing operations to the new coordinator before it falls into the sleeping mode. That includes routing packets (if the transition occurs during the routing operation), the routing caching table containing uplink and downlink nodes, etc. The information is encrypted by their pairwise shared key. By doing this, it will not effect to the existing dissemination flows.

### 5.4 Inspecting System

One of the most security concerns in sensor networks is node compromise. Due to resource limitation, sensor nodes are not equipped with any tamper-resistant hardware. Once a node is compromised, the adversary can extract all information stored in that node including all key materials. Then the adversary can use this node to perform various types of attacks to the networks. Defending against node compromise in routing is a non-trivial task.

**Figure 7.**
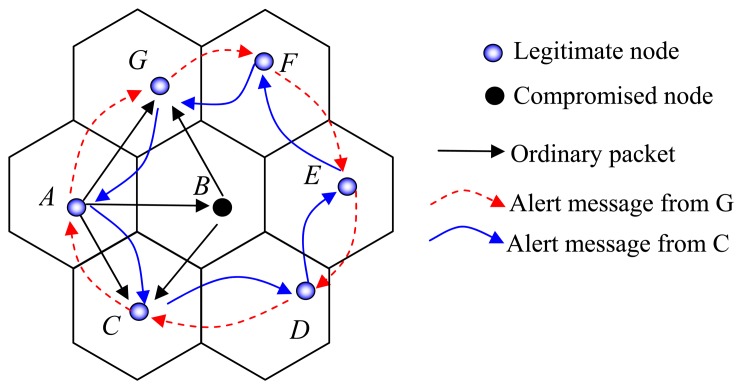
Inspecting System.

We propose an *inspecting system* which utilizes neighboring coordinators to detect if a node performs any wrongdoing. For each coordinator, there are six inspection nodes, called inspectors, which are neighboring coordinator of that node. For example, in Figure 7 there are six inspectors *A*, *C*, *D*, *E*, *F*, and *G* around node *B*.

If a compromised node modifies routing information such as *ID_S_*, *CID_S_*, *ID_Sink_*, *CID_Sink_*, *CID_This_* in the message, that can be easily detected by the uplink and downlink nodes because those nodes also maintain the source, sink, and information about its neighbors.

In case the compromised node *A* modifies *CID_Next_* and then relays to a wrong cell, the inspectors can detect this by overhearing the message sent out of *A* and computing next cell ID based on the information in the message. If the actual next cell ID is not the same as next cell ID in the message, then the inspectors will report this as a problem.

If *A* drops the packet, then the next cell node (uplink node) (which is also one of the inspectors) will detect by looking at packet sequence number. If *A* tries to modify the message content, it can be recognized by six next cell nodes or the source/sink. For example, if packet ID (*PID*) was modified, then the uplink node can detect that by verifying MAC using the pair-wise shared key *K_SA_*; if encrypted data {*DATA*}*K_S_* is modified, then the source/sink can detect by verifying the MAC with the source key *K_S_*.

Once the compromised node is detected, the inspectors alert to other coordinators and eliminate that node from participating in the routing process. Coordinators consider that cell a void cell and establish another route by finding a round path. For example in Figure 7, the coordinator *A* is legitimate node, and *B* is a compromised node. Node *C* and *G* are common inspectors of *A* and *B*, so they can receive the message sent out from *A* and *B* and can detect if *B* is doing something wrong. When *C* and *G* detect *B* as a compromised node, they send an alert message to the neighboring coordinator of *B* including *A*, *D*, *E*, and *F*. The alert message sent from *C* will go through *C*→*D*→*E*→*F*→*G*→*A*→*C*. The alert message sent out from *G* will go through *G*→*F*→*E*→*D*→*C*→*A*→*G*. This duplicated alert message makes sure that the compromised node is detected by two inspectors. The alert message is encrypted by the pairwise key between the sender and the receiver. For example, *G* sends an alert message to *F*:

(3) $$G\to F:ID_{G},CID_{G},ID_{B},CID_{B},\text{MAC}(K_{GF},ID_{G},CID_{G},ID_{B},CID_{B})$$

where *ID_G_*, *CID_G_* is node ID and cell ID of the inspector which detects compromised node, and *ID_B_*, *CID_B_* is the node ID and cell ID of the compromised node.

## 6. Analysis

In the previous version [12], we have argued that the proposed routing protocol is secure against common attacks mentioned in Section 2: spoofed, altered, or replayed routing information [9], [16], selective forwarding [16], sinkhole [16], *Sybil* [10], wormhole [11], *HELLO* flood (unidirectional link) attacks [16]. In this section, we are focusing on security of inspecting system, security and efficiency of the enhanced key management scheme.

### 6.1. Inspecting System Security

An adversary can target inspection nodes in two ways. Firstly, by deploying several nodes to report fake inspection information to the network. Secondly, by compromising a number of nodes to fool the entire network. We shall discuss those vulnerabilities in the following sections.

#### 6.1.1. The adversary deploys some nodes to report wrong inspecting information

Once a coordinator detects a compromised node, it sends an alert message to the neighboring coordinators of that node using a pairwise key. For example in equation (3), node *G* detects *B* as a compromised node, it sends an alert message to node *F* using pairwise key *K_GF_*. These keys are unknown to the adversary. So even she deploys some nodes and they send the wrong information, it can be easily detected by verifying MAC value in the alert message.

#### 6.1.2. The adversary compromises some inspectors to fool the entire network

Inspecting system is based on mutual inspection. This means that every coordinator could become an inspector, and vice versa. If the adversary compromises an inspector, then that inspector can be detected by other legitimate nodes. Therefore, the adversary cannot cheat the entire network unless she compromises all the nodes, which is not feasible.

### 6.2. Network Connectivity

In this section, we discuss network connectivity including local connectivity and global connectivity. Local connectivity is the probability a node could connect with neighbor nodes within its transmission range. Global connectivity is the ratio of the number of sensor nodes forming the largest isolated connected component in the final key graph *G* to the size of the whole network. In node compromise attacks, adversaries usually launch node compromise attacks in order to eavesdrop secure channels in the network, or using key materials revealed from compromised nodes to perform node replication attacks. In this regard, we discuss whether nodes compromised attacks could be used for eavesdropping or not.

We denote *A*(*n_i_*, *n_j_*) as *n_i_*, *n_j_* are neighboring, *B*(*n_i_*, *n_j_*) as the two nodes share a pairwise key. The local connectivity could be calculated as.

$$P_{\text{local}}=P(B(n_{i},n_{j})|A(n_{i},n_{j}))=\frac{P(B(n_{i},n_{j})\cap A(n_{i},n_{j}))}{P(A(n_{i},n_{j}))}$$

Probability that a node *n_i_* ∈ *G_i_* is a neighbor of node *n_j_*(*x_j_*, *y_j_*) is the integral of pdf *f*(*n_i_*) over the circle around node *n_j_* with radius *R*

$$P(n_{j}(x_{j},y_{j}))=\underset{G_{i}\left\|\left(x,y),(x_{j},y_{j}\right)\right\|\leq R}{\iint f(n_{i}(x,y))\text{dxdy}}$$

Because *n_j_* distribute in group *G_j_* following (1) the probability that *n_i_* ∈ *G_i_* is a neighbor of *n_j_* ∈ *G_j_*:

$$P(A(n_{i},n_{j})∥G_{i},G_{j})=\iint_{G_{j}}P(n_{j}(x_{j},y_{j}))f(n_{j}(x,y))\text{dxdy}$$

Hence,

$$P(A(n_{i},n_{j}))=\sum_{G_{i}\in \psi }\sum_{G_{i}\in \psi }P(n_{i}\in G_{i})P(n_{j}\in G_{j})P(A(n_{i},n_{j})∥G_{i},G_{j})$$

Denoted *S*(*G_i_*) is set of neighboring groups of *G_i_* we have

$$P(B(n_{i},n_{j})\cap A(n_{i},n_{j}))=\sum_{G_{i}\in \psi }\sum_{G_{i}\in S(G_{i})}P(n_{i}\in G_{i})P(n_{j}\in G_{j})P(A(n_{i},n_{j})∥G_{i},G_{j})$$

Because a sensor node is chosen in a given group with an equal probability, we have the local connectivity can be calculated as

$$P_{\text{local}}=\frac{\sum_{G_{i}\in \psi }\sum_{G_{i}\in S(G_{i})}P(A(n_{i},n_{j})\|G_{i},G_{j})}{\sum_{G_{i}\in \psi }\sum_{G_{j}\in \psi }P(A(n_{i},n_{j})\|G_{i},G_{j})}$$

Denoted *d* = *a* × *σ* is the distance between two deployment points of two neighboring cells, where *a* is a predetermined value. This *d* value has impacts on the local connectivity and global connectivity in the network. If the deployment distribution follows *Gaussian* distribution, then 99.87% nodes of a group reside within range of 3σ from its deployment point. Therefore, if the value *d* is much larger than 6σ, almost every nodes in a group reside in its cell area, and the neighboring nodes are from its own group. In this case, the local connectivity is very high, but the network is totally partitioned into isolated components, meaning global connectivity is very low. In case of the value *d* is smaller, the local connectivity may be low, but the global connectivity is high. So, choosing suitable value of *d* affects the network connectivity.

**Figure 8.**
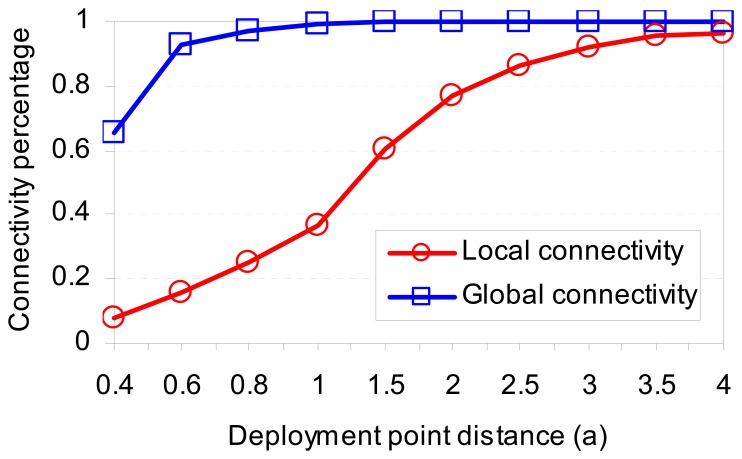
Network connectivity vs. Deployment point distance (*a*).

In the simulation, we change different values of *d* according to *a*. The simulation was carried out with 10,000 nodes in a network area of 2,000m×2,000m. The sensor communication range is set to 250m. The ratios of local connectivity and global connectivity also have various values as shown in Table 2 and Figure 8.

Note that, the connectivity is not affected by the number of nodes in a cell. Therefore, it does not matter whether nodes are in active or in sleeping mode, because each cell always maintains one coordinator.

When the distance between two deployment points of two neighboring cells is too low (*a* = 0.4; 0.6; 0.8 or 1.0), at any node *A*, there are many nodes of non-neighbor cells distributed around it. These nodes do not share any key-space with node *A*. So the local connectivity and global connectivity are reduced.

From Table 2, it is easy to see that our model gains high local and global connectivity when choosing suitable value of deployment point distances. With value *a*=1.5, the global connectivity is 0.9990, meaning that only 0.01% number of nodes in the network are waste.

### 6.3. Communication and Memory Overhead

The network lifetime is a critical goal in designing protocols for wireless sensor networks. In our proposal, we minimized the broadcast data requirement in establishing direct key between neighboring nodes. Our *1-hop* broadcast message length is *sizeof*(*ID*) *+ sizeof*(nonce) + *3 × sizeof*(*hash*). Comparing with other models in [30], the broadcast messages in key discovery phase contain hundreds of key, to achieve high connectivity. With CPPS in [13], the length of broadcast messages is *sizeof(ID)* + *5 × sizeof (polynomial ID)*, which is higher than ours.

The memory size for storing key materials derived from polynomials is *M = 3×(t + 1) log_2_q + sizeof* (*ID*) *+ sizeof* (nonce) (*bits*). This value, along with the number of nodes sharing a polynomial, affects to the resilience against node compromise attacks. This issue will be discussed in more detail in the following section.

### 6.4. Resilience against Node Compromise Attacks on Key Management

The proposed model has two stages that could be a target for node capture attacks. The first stage is a period between the distribution of sensor nodes to the target field, and establishment of pair-wise key. The second one is after broadcasting discovery messages and forming a connected pair-wise key network. The properties of two stages lead to different security levels.

In the first stage, adversaries can capture enough sensor nodes in a cluster to interpolate polynomials, and then eavesdrops KSDM messages to get *nonce* values. The number of sufficient compromised sensor nodes depends on the polynomial degree that is the memory for storing key materials. In this manner, attackers could generate all pair-wise keys in the cluster. So the security in this stage needs some consideration. But we need to remember that the time of this stage is quite short right after deployment, so a chance for an adversary to launch attacks is not highly possible.

In the second stage, from (2) we can see that each pair-wise key depends not only on key-space information but also on random values associated with each node. Suppose that a node *A* is compromised and an adversary could get all information stored inside the *A*'s memory. In this case, she could only get pair-wise keys between *A* and its neighbors, and a fraction of polynomial *f*(*N_A_*, *y*). Even if she compromises a sufficiently large number of nodes like *A*, then still it is not possible for an adversary to expose pair-wise key between non-compromised nodes *B* and *C*. This is due to the fact that the adversary does not have the information about the random numbers stored on the non-compromised nodes. So, compared with other schemes such as [26]-[30], our scheme has not been affected by node capture attacks on the links between non-compromised nodes.

## 7. Performance Evaluation

We have evaluated SCODE*plus* performance and compared with the SCODE [12], TTDD [17], and DD [18]. The evaluation was carried out by simulation on SENSE simulator (Sensor Network Simulator and Emulator) [20].

### 7.1. Simulation Model

The network comprises 400 nodes randomly deployed in a 2,000 m × 2,000 m area. We use the same energy model in *ns-2.1b8a* [21] that requires 0.66W, 0.359W and 0.035W for transmitting, receiving and idling, respectively. We set the power consumption rates of RC5 according to [22][23] for encryption, MAC computation, and random number generation are 0.65W, 0.48W, and 0.36W, respectively. As analyzed in [19][24], we set the time consumption for encrypting 64 bits with RC5 0.26 ms, generating 64 pseudorandom bits takes 0.26 ms, and computing a 4- byte MAC requires 0.13 ms. The simulation uses MAC 802.11 *Distributed Coordination Function* (DCF) and nominal transmission range of each node is 250m. *Two-ray ground* [25] is used as the radio propagation model.

Each data packet has 64 bytes, query packets and the others are 36 bytes long. Additional bytes for MACs and *nonce* values are also put into each message. The default number of sinks is 8 moving with speed 10m/s according to *random way-point* model. Two sources generate different packets at an average interval of 1 second. Summary of parameters and defined values are shown in Table 3.

### 7.2. Simulation Results

Since SCODE*plus* is targeted to multiple and mobile sinks in sensor networks, we study the impact of the number of sinks, sink's speed, and the density of the network. We measure the energy consumption, average delay (average response time to users), and success ratio (total number of packets has been delivered successfully).

#### 7.2.1. Impact of Sink Number

As many users may simultaneously access the sensor network, it is important to consider the impact of the sink number. For the sensor network area of 2,000 m×2,000 m, we set the number of sink varying from 1 to 8. Each sink moves with the maximum speed 10 m/s and a 5-second pause time. The number of total nodes and the number of sources are not changed.

**Figure 9.**
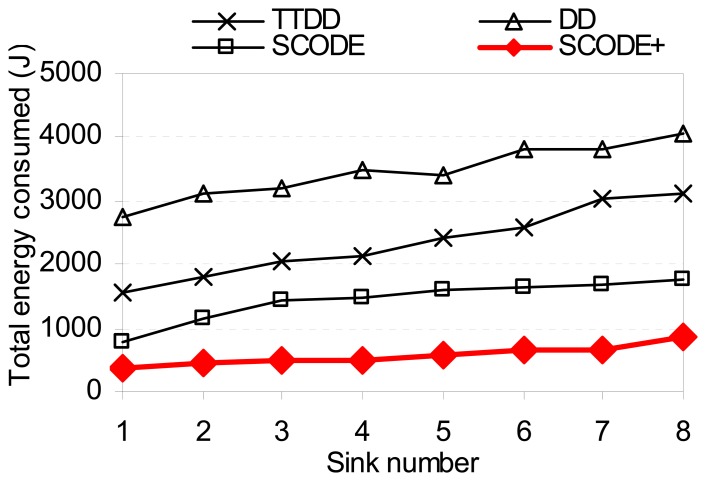
Energy consumption vs. sink number.

Figure 9 shows total energy consumption of SCODE as the number of sinks varies from 1 to 8. It demonstrates that SCODE*plus* is more energy efficient than SCODE, TTDD, and DD. This is because of three reasons. First, SCODE*plus* and SCODE are based on a coordination network, so that nodes in each cell negotiate among themselves to turn off its radio to significantly reduce energy consumption. Meanwhile, TTDD and DD must turn on all nodes to participate in routing.

Second, SCODE*plus* and SCODE optimize a number of transmission hops between sources and sinks that is based on the cell size to maximize the communication distance between two adjacent cells. Third, SCODEplus is based on the hexagonal network topology. Compared with square-grid used in SCODE, the hexagon decreases the number of cells, reduces transmission hops, and increases the number of sleeping nodes to save energy. Also, using *Gaussian* network deployment can reduce the number of void cells, thus reduce the number of round paths compared with SCODE.

**Figure 10.**
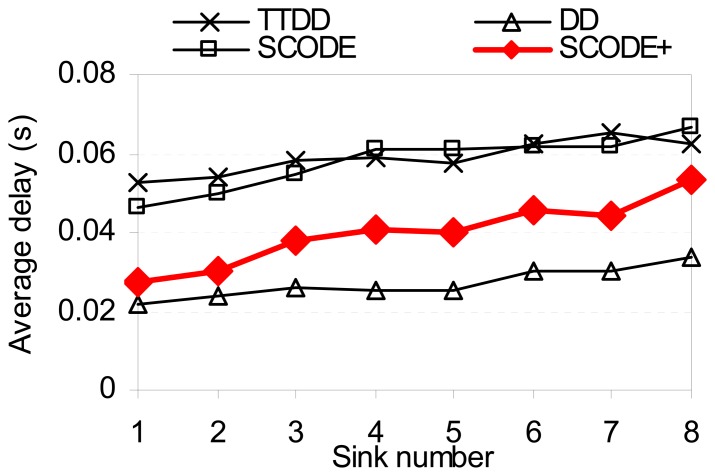
Average delay vs. sink number.

**Figure 11.**
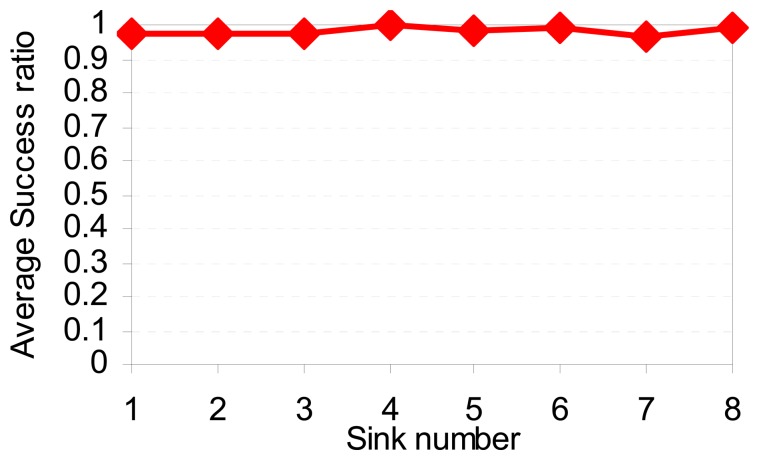
Success ratio vs. sink number.

Figure 10 plots the average end-to-end delay of SCODE. The figure shows that the delay of SCODE*plus* is mush smaller than SCODE. Figure 11 shows that the success rate of SCODE*plus* is always above 97 percent. It means that SCODE*plus* delivers most of packets successfully.

#### 7.2.2. Impact of Sink Mobility

In order to see the impact of sink mobility, we ran the simulations for different sink speeds (0 to 30m/s). In this experiment, the network consists of 8 mobile sinks and 400 sensor nodes. The number of sources does not change.

**Figure 12.**
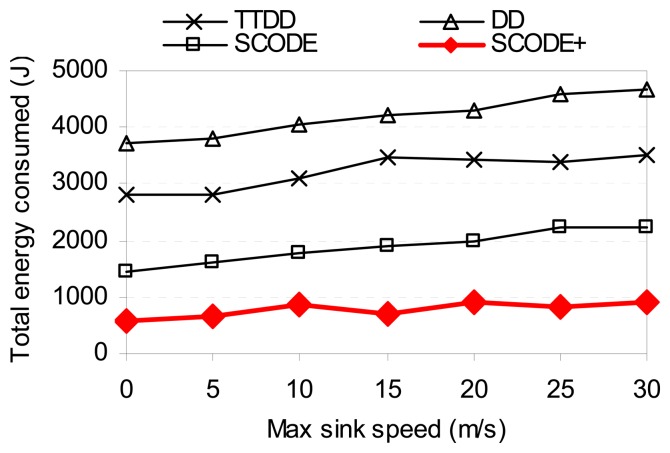
Energy consumption vs. sink speed.

Figure 12 shows the energy consumption as the sink speed changes. In both low and high speeds of the sinks, SCODE*plus* shows that the total energy consumed is much less than SCODE, TTDD, and DD. The reason is because, aside from above reasons, SCODE*plus* reduces the number of re-transmissions of query and up dating sink's locations while the sinks are moving. The query only needs to resend as the sink moves to another cell. In contrast, TTDD and DD send more messages to propagate new location of the sinks throughout the sensor field to all sensor nodes.

**Figure 13.**
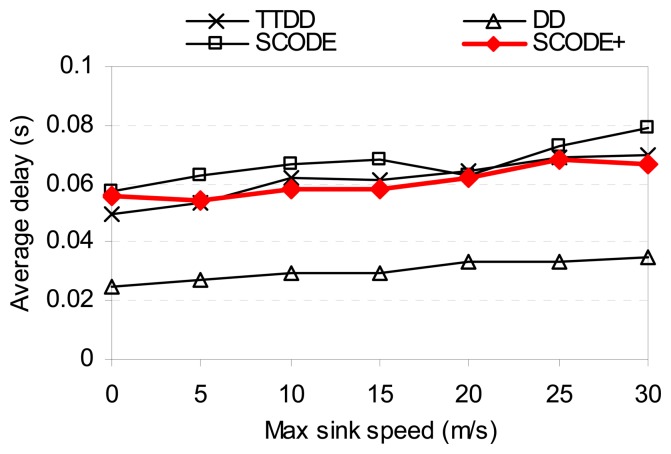
Average delay vs. sink speed.

**Figure 14.**
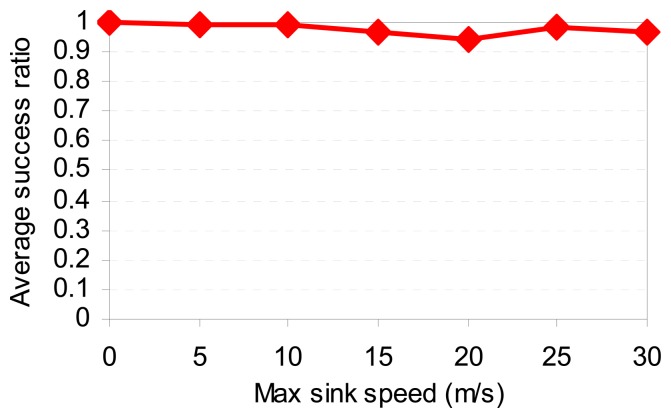
Success ratio vs. sink speed.

Figure 13 shows the delay of SCODE*plus*. Compared with the result in Figure 10, the average delay performance of SCODE*plus* drops as the sinks are moving. This is because when the sinks move to another cell, they have to send a removal message to clear the old dissemination paths, and then re-send a new query to establish a new one. Doing this causes more delay compared with the stationary scenarios. In Figure 14, as the sinks speed up, the average success ratio is always above 97%. This results show that SCODE*plus* handles mobile sinks efficiently

#### 7.2.3. Impact of Node Density

To evaluate impact of node density on SCODE*plus*, we vary the number of nodes from 200 to 600. The number of sinks is 8. Each sink keeps moving with speed 10m/s as the default setting. The number of sources is 2. The sensor field size is not changed.

**Figure 15.**
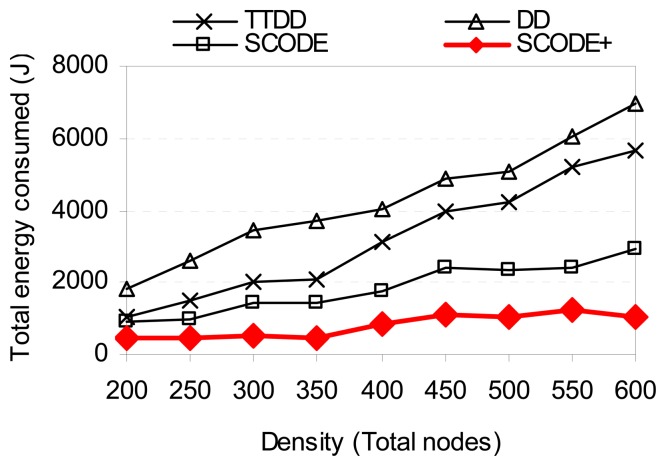
Energy consumption vs. sink speed.

**Figure 16.**
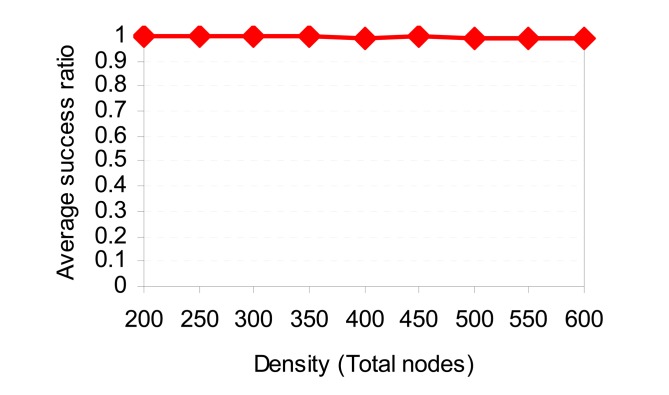
Success ratio vs. sink speed.

Figure 15 shows the energy consumption with different node densities. The figure demonstrates that SCODE*plus* consumes less energy than SCODE, TTDD, and DD. As the number of nodes increases, the total energy consumed slightly increases while that of TTDD and DD significantly increases. This is mainly because SCODE*plus* turns off radio most of the time. Therefore, energy is consumed mostly by the coordinators. Whereas, in TTDD and DD, nodes do not participate in communication still consume much energy in idling mode. Figure 16 shows that SCODE*plus* almost delivers all packets successfully (above 99%).

## 8. Related Work

Research on the sensor network routing has been carried out for nearly a decade. Heinzelman [1] introduce a clustering algorithm for WSNs, called LEACH. In LEACH, sensors are organized into clusters. Each cluster has one cluster head (CH) which collects and aggregates information from its members (non-CH sensors in the same cluster) and passes on information to the base station (BS). However, LEACH has a number of shortcomings. LEACH assumes every node can directly reach the base station by transmitting with sufficiently high power. However, one-hop transmission directly to the base station is not feasible in large-scale WSNs due to the resource-limitations of sensors. On the other hand, LEACH is vulnerable from several attacks including HELLO flood, selective forwarding, and Sybil attacks [16].

Recent works have taken into account of both performance and security such as SecRout [2], SEEM [3], SeRINS [4], and TTSR [17]. SecRout [2] guarantees that sinks receive correct queries resulted from the sensor network. Only a high efficient symmetric cryptography is used in SecRout. SEEM [3] uses a principle similar to the Client/Server software architecture. The BS performs the route discovery and maintenance as well as route selection. Instead of a single path, the BS periodically selects a new path from multi-paths based on the current energy level of nodes along each path. SeRINS [4] focuses on detecting and isolating compromised nodes. The major shortcoming of those approaches is that they assume the BS is stationary and all sensor nodes know the BS's location. This assumption makes them fail to work in case the BS (sink) is mobile. TTSR [17] is a secure and efficient routing protocol for heterogeneous sensor networks (HSNs) which takes advantage of powerful high-end sensors (H-sensors) in an HSN. TTSR possesses a similar sink mobility problem with SecRout, SEEM, and SeRINS. On the other hand, TTSR is only suitable for HSNs with sufficient powerful sensor nodes, not large-scale homogeneous WSNs. Besides, relying only on some particular nodes makes them prone to deplete there energy sooner or later. Using fixed coordinators makes attackers easy to choose ‘right’ nodes to compromise.

There are a number of routing protocols aiming to support mobile sinks in WSNs [5][6][7][17][18]. Declarative Routing Protocol [7], and Directed Diffusion (DD) [18] suggest that each mobile sink needs to continuously propagate its location information throughout the sensor field, so that all sensor nodes get updated with the direction of sending future data reports. However, frequent location update from multiple sinks leads to both increased collisions in wireless transmissions and rapid power consumption of the sensor's limited battery supply. SAFE [5] uses flooding that is geographically limited to forward the query to nodes along the direction of the source. Considering the large number of nodes in WSNs, the network-wide flooding may introduce considerable traffic. SEAD [6] considers the distance and the packet traffic rate among nodes to create a near-optimal dissemination. SEAD strikes a balance between end-to-end delay and power consumption that favors power saving over delay minimization. It is therefore only suitable for applications with a less strict delay requirement. TTDD [17] exploits local flood within a local cell of a virtual grid which sources build proactively. However, it does not optimize the path from the source to the sinks. When a source communicates with a sink, the restriction of grid structure may multiply the length of a straight-line path by $\sqrt{2}$. Also, TTDD frequently renews the entire path to the sinks. It therefore increases energy consumption and connection loss ratio. Those multi-hop routing protocols face many security problems from spoofed, altered, or replayed routing information [9][16], selective forwarding [16], sinkhole [16], Sybil [10], wormhole [11], and HELLO flood (unidirectional link) attacks [16].

Our protocol overcomes those shortcomings by considering security and routing protocol at the design time. The scheme is based on GAF, along with a hexagonal deployment model to achieve better security and efficiency. It securely disseminates 97% data packets to the mobile sinks successfully and even more efficiently than non-secure approaches like TTDD and DD.

## 9. Conclusions

In this paper we have presented a first, novel energy-efficient secure routing and key management scheme for mobile sinks in sensor networks, namely SCODE*plus*. It is a significant enhancement of our previous study, *Secure COodination-based Data dissEmination protocol for mobile sinks* (SCODE) [12]. Besides several improvements, this study was recognized by a careful consideration of security (key management scheme) during the design time. Those not only enhance security but also increase efficiency of the proposed scheme.

SCODE*plus* takes advantages of a coordination network based on GAF and a hexagonal deployment model to apply an energy-efficient secure routing and key management scheme for mobile sinks. In SCODE*plus*, the sensor network plane is partitioned into a hexagonal structure. An efficient key management based on Blundo's scheme is applied to reduce memory requirements and increase the security level. The cell size is optimal to increase network connectivity and reduce the number of transmission hops. Sensor nodes in each cell negotiate among each other so that only one node, called coordinator, stays awake and the others fall into sleeping mode for the sake of energy. After an interval, all nodes wake up and re-elect a new coordinator. By doing this, nodes do not quickly run out of its energy, thus prolonging the network lifetime. Communication between sources and sinks are established via coordinators and the shortest path is calculated.

Through analysis, we show that SCODE*plus* can defend against several common attacks in the sensor network routing including spoofed routing information, selective forwarding, sink-hole, worm-hole, Sybil, and HELLO flooding attacks. The enhance key management in this version can perfectly eliminate the impacts of node compromise attacks on links between non-compromised nodes which most existing key management schemes have faced. Our simulation results have shown that SCODE*plus* achieves much better performance compared with our previous scheme SCODE as well as existing ones, *Two-Tier Data Dissemination* (TTDD) [17] and *Directed Diffusion* (DD) [18]. We have shown that SCODEplus reduces much more energy consumption, delay time, and increases the success ratio (always above 97%, which means SCODE*plus* delivers most packets to mobile sinks successfully).

## Figures and Tables

**Table 1.** Notations.

**Notation**	**Description**
*ID_A_*	Identification of node A
*CID_A_*(*X*, *Y*)	Cell identification of node A, indicating X and Y axes of the cell
*PID*	Packet Sequence Number (packet ID)
*R*	Radio range of a sensor node
*r*	Cell size
*K_A_*	A secret key held by node A
*K_ab_*	A shared key between A and B.
MAC (*K*, *M*)	Message authentication code of message M using a symmetric key *K*
{*M*}K	Message M is encrypted with a key *K*
*N_0_*, *N_1_*	*Nonces*, one-time random number generated by nodes
*A* ➔ broadcast: *M*	Node *A* broadcasts a message *M*
*A* ➔ *B*: *M*	Node *A* sends a message *M* to node *B*

**Table 2.** Network connectivity.

***a***	**Local connectivity**	**Global connectivity**
0.4	0.0787	0.6546
0.6	0.1577	0.9290
0.8	0.2524	0.9704
1.0	0.3643	0.9921
1.5	0.6036	0.9990
2.0	0.7720	0.9994
2.5	0.8617	0.9998
3.0	0.9226	0.9999
3.5	0.9555	0.9999
4.0	0.9657	1

**Table 3.** Simulation parameters and defined values.

**Simulation Parameters**	**Value**
*N* (total nodes)	400 nodes
*A* (network size)	2,000 m × 2,000 m
Transmission	0.66W
Reception	0.359W
Idle	0.035W
RC5 encryption of 64 bits	0.26 ms
Pseudorandom number generation	0.26 ms
4-byte MAC generation	0.13 ms
*R*	250 m
Data packet size	64 bytes
Other packet size	32 bytes
Number of mobile sinks	8
Default speed of mobile sinks	10 m/s
Data generation interval	1 second
